# Pneumonic Plague Transmission, Moramanga, Madagascar, 2015

**DOI:** 10.3201/eid2303.161406

**Published:** 2017-03

**Authors:** Beza Ramasindrazana, Voahangy Andrianaivoarimanana, Jean Marius Rakotondramanga, Dawn N. Birdsell, Maherisoa Ratsitorahina, Minoarisoa Rajerison

**Affiliations:** Institut Pasteur de Madagascar, Antananarivo, Madagascar (B. Ramasindrazana, V. Andrianaivoarimanana, J.M. Rakotondramanga, M. Rajerison);; Northern Arizona University, Flagstaff, Arizona, USA (D.N. Birdsell);; Ministry of Public Health, Antananarivo (M. Ratsitorahina)

**Keywords:** *Yersinia pestis*, pneumonic plague, transmission, Madagascar, outbreak, bubonic plague, vector-borne infections, bacteria

## Abstract

During a pneumonic plague outbreak in Moramanga, Madagascar, we identified 4 confirmed, 1 presumptive, and 9 suspected plague case-patients. Human-to-human transmission among close contacts was high (reproductive number 1.44) and the case fatality rate was 71%. Phylogenetic analysis showed that the *Yersinia pestis* isolates belonged to group q3, different from the previous outbreak.

Plague, caused by the bacterium *Yersinia pestis*, is a fleaborne disease responsible for the death of tens of millions of persons throughout history (*1**,**2*). The bacterium spread to Madagascar in 1898 through trade routes and became endemic in the country’s central highlands (*3*). Despite considerable control efforts, *Y. pestis* remains a public health threat in Madagascar (*4**–**6*). For the past 2 decades, Madagascar reported the highest number of human infections in the world (*7*).

Bubonic plague is the most common disease form resulting from the bite of an infected flea. As bubonic plague worsens, it can progress to pneumonic plague, in which persons expel infectious, aerosolized respiratory droplets. During pneumonic plague outbreaks, person-to-person transmission facilitates the spread from the initial infected person to family members and the wider community (*5**,**8*). Pneumonic plague is rare but poses a substantial danger in Madagascar because of the country’s relatively weak healthcare system (*4**,**6*) and widespread traditional burial methods involving interaction with corpses. Understanding how pneumonic plague outbreaks spread is vital for managing disease prevention and educating the population. We investigated the transmission chain of a pneumonic plague outbreak that occurred in Madagascar outside the normal plague season (October–March) (*9*) in a remote area that had been free of human plague for 13 years.

## The Study

**Figure 1:**
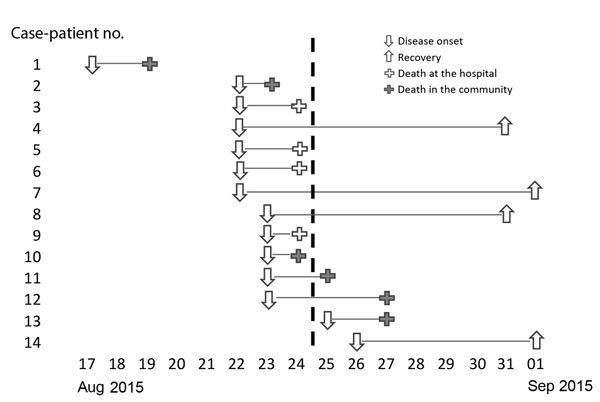
Course of pneumonic plague outbreak in Moramanga, Madagascar, August 17–September 1, 2015 (N = 14). Each line corresponds to a case-patient and describes disease outcomes. The vertical dashed line denotes when control measures began.

Over a 10-day period, 14 persons became ill with pneumonia and fever. The first case-patient (case-patient 1) displayed symptoms on August 17, 2015, while in a remote area in Antsahatsianarina hamlet, Tsiazompody village, Ampasipotsy Gara commune, within the Moramanga district of Madagascar (Technical Appendix Table). Case-patient 1 was a 22-year-old man who had traveled 1 week before disease onset. After returning home, he experienced chest pain, fever, and cough. Two days later, his condition deteriorated and, while relatives and neighbors assisted him to the nearest health center, he died near Beravina, Madagascar. He was buried near Beravina in a traditional manner with a 2-night wake, exposing the family and community to the pathogen and initiating a chain of transmission. On August 22 and 23, plague symptoms developed in members of case-patient 1’s immediate family (N = 2), his extended family (N = 6), and the community (N = 3). Of these, 4 died, and 7 self-referred to the Moramanga district hospital, where 4 died upon arrival, and 3 survived after a 6- to 9-day course of antimicrobial drug treatment. On August 25, a second chain of transmission (N = 2) was identified (Figure 1). One case-patient was from the outbreak epicenter, and the other was a suspected nosocomial transmission; no connection to the other cases could be found except occupying the same hospital space as some of the earlier case-patients. Transmission ceased with these last 2 case-patients.

**Figure 2:**
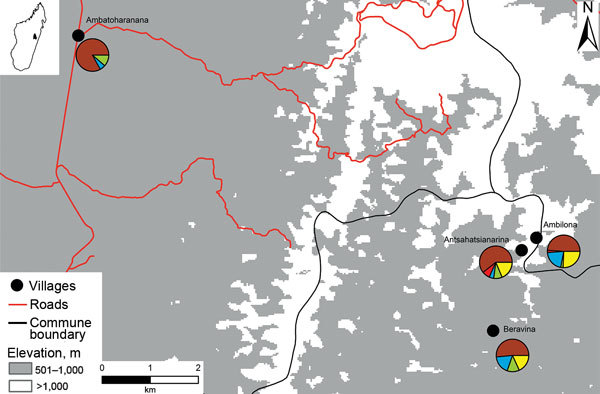
Map of pneumonic plague outbreak (n = 14) in the commune of Ampasipotsy Gara in Moramanga, Madagascar, 2015. The index case-patient (case-patient 1) was infected with *Yersinia pestis* at Antsahatsianarina and spread the bacterium to Beravina (burial site of case-patient 1), Ambilona (case-patient 10’s home), and Ambatoharanana (burial site of case-patient 2 and home of case-patient 14). Each pie chart indicates the proportions of plague cases (red), seropositive contacts (blue), seronegative contacts (green), unsampled contacts (yellow), and noncontacts (brown) among the total inhabitants of each site. Pie chart details are given in the Technical Appendix).

Before illness onset, case-patients 2–14 had social or spatial contact with a symptomatic person who could be traced back to case-patient 1. Two of the deceased case-patients (2 and 10) were visitors to Antsahatsianarina who had returned home to their respective communities Ambatoharanana and Ambilona (Figure 2), potentially spreading the bacterium to others. To investigate the extent of pathogen spread, we identified 123 case-patient contacts in 4 communities connected with case-patient 1 (Technical Appendix Table). On August 24, 2015, an outbreak investigation protocol was applied by the Institut Pasteur de Madagascar and the Malagasy Ministry of Health, whose ethics committee approved the study (068-MSANP/CE). Verbal consent was obtained from 71 contacts to test their serum for plague antibody with a capsular antigen fraction 1 (F1) IgG ELISA (*10**,**11*); 7/20 (35%) contacts from Antsahatsianarina, 12/20 (60%) from Beravina (the burial site of case-patient 1), 9/10 (90%) from Ambilona, and 7/21 (33%) from Ambatoharanana were seropositive (Figure 2; Technical Appendix Table). The 35 contacts positive for F1-specific antibody were given chemoprophylaxis, and their infections remained subclinical.

The 14 case-patients who had sought medical care for pneumonia and fever had clinical signs and symptoms consistent with those for plague. To confirm *Y. pestis* infection, we performed bacteria culture on sputum or organ puncture (*12*) and serologic testing of serum samples (*10*). According to the international standards definition (*13*), 4/5 patients (1 deceased) were confirmed positive for *Y. pestis* (2 by culture and 2 by seroconversion) and 1/5 was presumptive for *Y. pestis* infection. No samples were collected from the other 9 (all deceased) persons, who were thus considered suspected plague case-patients (*13*). 

More men (n = 10) than women (n = 4) were infected. Median patient age was 22.5 (range 15–80) years, and the overall case-fatality rate was 71%. The most common signs and symptoms were fever, dyspnea, chest pain, and cough (100% of patients); blood-stained sputum (93%); chills (86%); and headache (71%). The average duration from onset of full-blown disease to death was 1.9 days, and the average infectious period was 3.5 days. Basic reproductive number (R_0_) was 1.44, and the transmission rate was 0.41 susceptibles/day (*14*).

To investigate the possible outbreak origin, we trapped 100 rats and mice over a period of 3 nights at Antsahatsianarina, Beravina, and Ambilona. Rodent spleens were sampled in accordance with the directive 2010/63/EU of the European Parliament (http://eur-lex.europa.eu/LexUriServ/LexUriServ.do?uri=OJ:L:2010:276:0033:0079:EN:PDF) and tested by using an F1 antigen rapid diagnostic test (*3*). A total of 22 (22%) rats, but no mice, from the 3 communities tested positive for plague antigen (Table).

**Table:** Number of *Yersinia pestis* antigen–positive rodents in a study of pneumonic plague transmission among humans, Moramanga, Madagascar, 2015*

Locality and species	No. samples	No. RDT positive	No. RDT negative
Antsahatsianarina			
* Rattus rattus*	73	16	57
* Rattus norvegicus*	10	1	9
* Mus musculus*	4	0	4
Beravina			
* Rattus rattus*	4	3	1
* Rattus norvegicus*	0	0	0
* Mus musculus*	2	0	2
Ambilona			
* Rattus rattus*	6	2	4
* Rattus norvegicus*	1	0	1
* Mus musculus*	0	0	0
Total	100	22	78

*A rapid diagnostic test (RDT) was used to test for *Yersinia pestis* antigen.

The lack of human plague activity in this area for the past 13 years suggests that a reservoir species, such as rodents or fleas, transmitted bubonic plague to case-patient 1 and that the disease later progressed into the pneumonic form. Relatives of case-patient 1 indicated that moribund rodents were present near his home 2 weeks before he became ill. The family who hosted case-patient 1 during his travels (1 week before his disease onset) reported no disease present in their household. Taken together, the epidemiologic data suggest that case-patient 1’s hamlet (Antsahatsianarina) was the probable outbreak epicenter. 

Using published single-nucleotide polymorphisms (*15*), we assigned isolates from 2 case-patients and 1 recently sampled rat to the *Y. pestis* q3 phylogenetic subgroup within group I (node k). The matched genetic grouping between the 2 human samples is consistent with human-to-human transmission. The matched genetic grouping among all 3 samples is consistent with *Y. pestis* in the initial case-patient originating from the environment rather than another human. We compared these phylogenetic data with data from 4 archived isolates (human and rodent) obtained in 2000, 2002, and 2003 from the same area. The archived isolates were also assigned to group I (node k), but unlike the isolates from this study, they were assigned to the q2 subgroup (*15*). This finding indicates that the recent outbreak did not arise from the same phylogenetic groups responsible for past outbreaks and illustrates how outbreaks in different years are probably conferred by *Y. pestis* from different environmental sources. As of January 2017, 4 lineages (q1, q2, q3, and q4) have been recorded in Moramanga, suggesting multiple genotypes are persisting within this region (*15*).

## Conclusions

Despite Madagascar having an effective surveillance system, plague control remains a public health challenge. Pneumonic plague is rare but persists as a threat in Madagascar, where poor healthcare systems and traditional burial practices promote these outbreaks. Our findings provide additional understanding of pneumonic plague transmission patterns, argues for continued public education, and informs authorities about effective outbreak response practices.

Technical Appendix Numbers of seropositive, seronegative, and unsampled contacts of plague case-patients among the total population per site in Moramanga, Madagascar in 2015.
