# Supplementary material for: Pneumonic Plague Transmission, Moramanga, Madagascar, 2015
Source: Emerg Infect Dis. 2017 Mar;23(3):521–4. doi: 10.3201/eid2303.161406 (PMC5382734; doi:10.3201/eid2303.161406)
Supplement: Technical Appendix — Numbers of seropositive, seronegative, and unsampled contacts of plague case-patients among the total population per site in Moramanga, Madagascar in 2015. [file 16-1406-Techapp-s1.pdf]

Article DOI: http://dx.doi.org/10.3201/eid2303.161406

Pneumonic Plague Transmission, Moramanga, Madagascar, 2015

Technical Appendix

Technical Appendix Table. Number of plague case-patients; seropositive, seronegative, and unsampled contacts among identified contacts; and total population numbers per site, Moramanga, Madagascar, 2015*

Locality	Village	Commune	Latitude	Longitude	Altitude, m	No. pop	CP no.	No. sero-positive	No. sero-negative	No. un-sampled	Rationale for testing
Antsahatsianarina	Tsiazompody	Ampasipotsy Gara	18°53.610'S	48°19.236'E	983	94	12	7	13	29	Outbreak epicenter (home of CP1 and other plague CPs)
Ambilona	Tsiazompody	Ampasipotsy Gara	18°53.480'S	48°19.479'E	1,018	21	1	9	1	11	Home of CP10, who was exposed to CP1
Beravina	Tsiazompody	Ampasipotsy Gara	18°54.524'S	48°18.916'E	943	34	0	12	8	12	Burial site of CP1
Ambatoharanana	Ambatoharanana	Ambatoharanana	18°51.282'S	48°13.944'E	906	102	1	7	14	0	Burial site of CP2, home of CP14 who was exposed at MDH

*CP, case-patient; MDH, Moramanga district hospital; pop, population.
